# Pharmacotherapy of Pain in the Older Population: The Place of Opioids

**DOI:** 10.3389/fnagi.2016.00144

**Published:** 2016-06-16

**Authors:** Milica Prostran, Katarina Savić Vujović, Sonja Vučković, Branislava Medić, Dragana Srebro, Nevena Divac, Radan Stojanović, Aleksandar Vujović, Lepa Jovanović, Ana Jotić, Nataša Cerovac

**Affiliations:** ^1^Department of Pharmacology, Clinical Pharmacology and Toxicology, Faculty of Medicine, University of BelgradeBelgrade, Serbia; ^2^Hospital for ENT, KBC Dragiša MišovićBelgrade, Serbia; ^3^Institute for Gerontology and Palliative CareBelgrade, Serbia; ^4^Clinic for Otorhinolaryngology and Maxillofacial Surgery, Clinical Centre of Serbia, Faculty of Medicine, University of BelgradeBelgrade, Serbia; ^5^Clinic for Neurology and Psychiatry for Children and Youth, Faculty of Medicine, University of BelgradeBelgrade, Serbia

**Keywords:** pain, opioids, older persons, aging, polypharmacy, adverse effect

## Abstract

Pain is a common symptom in older people. It is possible that pain is underreported in older persons due to an incorrect belief that it is an inevitable part of aging. Opioid analgesics are potent medications, with confirmed efficacy for the treatment of moderate to severe pain. These drugs are commonly used in older persons. However, there is insufficient evidence regarding safety of opioids in older patients. One of the reasons for this is the lack of randomized, controlled clinical trials. People of advanced age often have comorbidites and use other prescription drugs, as well as over-the-counter (OTC) compounds, thus making them more suceptible to the risk of interactions with opioids. Significant pharmacokinetic and pharmacodynamic changes that occur with advancing age increase the risk of adverse effects of opioids. There are also some discrepancies between guidelines, which recommend the use of lower doses of opioids in older patients, and the findings in the literature which suggest that pain is often undertreated in this age group. It seems that there are significant variations in the tolerability of different opioid analgesics in older people. Morphine, fentanyl, oxycodone, and buprenorphine are still the preferred evidence-based choices for add-on opioid therapy for these patients. However, the safety and efficacy of other opioids in older patients, especially if comorbidities and polypharmacy are present, is still questionable. This review addresses the most important aspects of the use of opioids in older persons, focusing on pharmacokinetics, pharmacodynamics, adverse effects, and interactions.

## Background

The population of older people is normally classified as being aged 65 years and above. The projections of the Administration of Aging are that by 2030 the population of people over the age of 65 will increase two-fold in the United States (Atkinson et al., 2013). The proportion of people aged 60 and over is also rising every year throughout Europe, including our country (Pergolizzi et al., 2008).

Pain is common in the older population. It is important to assess the pain, treat, and evaluate it. Also, it is important to recognize and address the side effects of analgesic drugs that can be very challenging due to the alterations in the pharmacokinetics of medications which occur with normal physiologic aging (Chau et al., 2008). The use of opioid analgesics in the treatment of moderate to severe pain in people of advanced age is additionally complicated by the presence of other diseases (comorbidities) and the use of other medications which increases the risk of possible interactions.

Pain may be underreported by older persons due to the incorrect belief that it is necessarily associated with aging. In patients with cancer pain, it is underreported because of the fear of disease progression. Therefore, the caregivers and relatives are sometimes a more reliable source of information (Ferrell et al., 1991).

The studies on the treatment of pain in older patients are sparse. Mercadante and Giarratano in their systematic review of differences between adults and older persons and between male and female patients in randomized clinical trials on pain identified only three studies that focused on the population of advanced age (Mercadante and Giarratano, 2014).

There is an increasing number of articles focused on pain assessment in patients with dementia (Warden et al., 2003). Managing pain in patients with dementia is a challenge. Dublin et al. (2015) in a prospective cohort study examined whether prescribing of opioid medications is associated with a higher risk of dementia or with greater cognitive decline. They concluded that people with the heaviest opioid or non-steroidal anti-inflammatory drugs (NSAIDs) use had slightly higher dementia risk than people with little or no use.

Older patients with dementia or during terminal phases of diseases are treated as vulnerable subjects. It is a special problem to conduct clinical trials with vulnerable subjects due to their (in)ability to understand and sign the informed consent. Informed consent is a process of communication between a patient and physician that results in the patient's permission or agreement to undergo a specific medical procedure (Prostran et al., 2012). It may be one of the reasons why so few randomized, controlled clinical trials on the use of opioids in older people are available.

## Opioid analgesics in older persons

Chronic pain occurs in 45–85% of the older population and the need to treat chronic pain is growing substantially. Unfortunately, the treatment of chronic pain is not always correctly targeted, which leads to a reduced quality of life, with decreased socialization, depression, sleep disturbances, cognitive impairment, disability, and malnutrition (Gianni et al., 2009).

Opioids are drugs for the treatment of acute and chronic, moderate to severe pain, such as nociceptive and neuropathic pain. These drugs belong to a class of drugs with a known efficacy in pain, in both young and older patients (Kahan et al., 2006; Kuehn, 2009).

Up to 76% of older people in the community experience pain (Abdulla et al., 2013). There are no gender differences among 65 to 80-year-olds in terms of global pain status (LeResche et al., 2015).

The American Geriatric Society suggests the use of opioids in older persons (American Geriatric Society Panel, 2009). It is recommended to start with paracetamol and add on opioids when needed to manage pain in a stepwise approach (Veal and Peterson, 2015). The decision as to which opioid should be added on to paracetamol is a difficult one due to a lack of evidence regarding safety and tolerability. Most of the clinical trials exclude the frail patients and/or minimally include older people. A retrospective analysis of a consecutive sample of patients with cancer admitted to a home care program was performed and it was shown that in the older population there was a lower consumption of opioids, the subcutaneous route for administration was used more frequently, and palliative sedatives were required less often (Mercadante et al., 2016).

World Health Organization recommends that the treatment of pain in Step 3 includes opioids such as morphine, hydromorphone, fentanyl, levorphanol, methadone, and oxycodone. For the effective and safe use of these drugs it is well to be familiar with opioid pharmacokinetics, equi-analgesic dosing, and adverse effects. Antiepileptic drugs, antidepressants, and local anesthetics as adjuvant analgesics have a role in the treatment of neuropathic pain. Adjuvant analgesics can enhance the effects of opioids, especially in cases where opioid responsiveness may be in question (Prommer and Ficek, 2012).

Patients with moderate to severe pain, pain related to functional organ impairment, or diminished pain-related quality of life should be considered for opioid therapy (low quality of evidence, strong recommendation; Kaye et al., 2010). Also, patients taking opioid analgesics should be reassessed often for attainment of treatment goals, adverse effects, and safe and responsible medication use (moderate quality of evidence, strong recommendation).

Previous studies have shown that older patients require lower doses of opioids. Studies comparing the efficacy and tolerability of opioids, such as fentanyl patches (Menten et al., 2002), morphine (Kaiko, 1980), and sublingual buprenorphine (Nasar et al., 1986) in the older population and other populations have shown that the older people respond, as well as, or even better, to opioid treatment than younger age groups. Additionally, patients who underwent hip or knee arthroplasty under general anesthesia were identified in a retrospective cohort study design; older patients received lower doses of opioids intraoperatively and were less likely to require rescue analgesia (Ladha et al., 2015). Despite responding well to lower doses of analgesics, it has been shown that a significant proportion of older persons, either at nursing homes or community-living, have inadequately addressed daily pain associated with cancer or other conditions (Bernabei et al., 1998; Landi et al., 2001).

## The efficacy of different opioids in the treatment of pain in older persons

Morphine has been used to treat malignant pain for many years and has been the subject of a large number of trials, generally involving small numbers of patients. Also, morphine has been used for the management of the persistent non-malignant pain too, often as a comparator to newer opioids where similar efficacy has been demonstrated (Lee et al., 2015). Morphine is metabolized in the liver. Morphine-6-glucuronide (M6G) is potent analgesic and morphine-3-glucuronide (M3G) may cause neuroexcitatory effects. These metabolites are excreted in bile and then feces and urine after the last dose is given. Accumulation of the metabolites in patients with renal impairment, may cause side effects requiring dose adjustment, or switching to an alternative opioid (Gianni et al., 2009).

Recently, in one study it was observed that liquid morphine reduced the chronic non-cancer pain in 10 older patients (with a mean age of 75.5 years and medical co-morbidities; Lee et al., 2015). Future larger and well-designed studies should focus on the use of liquid morphine for older people. OROS hydromorphone (Jurnista) is a once-daily, extended-release formulation that uses the OROS push-pull technology to provide controlled release of the semi-synthetic opioid hydromorphone (Lussier et al., 2010). According to Lussier et al. (2010), once daily usage has an advantage over longer-acting formulations which are used twice-daily (Lussier et al., 2010).

A combination of morphine and gabapentin produces better analgesia than the individual drugs or placebo (Mao et al., 2011).

Similar efficacy to newer opioids, such as oxycodone, fentanyl, and methadone has been demonstrated (van Ojik et al., 2012).

Oxycodone is used in different types of surgical procedures and patient groups, from preterm newborns to older patients (Kokki et al., 2012). Variability of different enteral drug formulations will decrease the need for intravenous patient-controlled analgesia but it will increase the use of transmucosal administration and enteral oxycodone-naloxone controlled-release tablets.

Fentanyl, as a short-acting synthetic pure opioid, offers an excellent option for the treatment of cancer and chronic pain (Panagiotou and Mystakidou, 2010). Intranasal fentanyl has been investigated to assess its potential as a well-tolerated acute postoperative breakthrough pain relief medication. It has been shown to be superior to oral transmucosal fentanyl for the treatment of cancer breakthrough pain. It had been reported for postoperative or acute pain treatment of children and adults in the pre-hospital and hospital settings, but it had not been investigated in people of advanced age.

Recent study by Likar et al. (2008) in patients with moderate and severe pain showed that transdermal buprenorphine benefited patients to a comparable or even higher extent in 65-year-olds compared with the younger age group, supporting the need to address the problems with underprescribing in this age group (Likar et al., 2008). In one prospective, observational, multicenter study of patients with various chronic pain conditions the safety, perceptions, and discontinuation of treatment with transdermal buprenorphine patches were assessed. In patients with arthritis or other musculoskeletal disorders this patches were “effective” or “very effective” at relieving pain and they were “satisfied” or “very satisfied” with this therapy (Serpell et al., 2015).

Tapentadol as a newer opioid, acts on both the mu receptors and on neuronal reuptake of noradrenaline. This drug has no significant analgesically active metabolites, which have some advantages, particularly in comparison with tramadol (Veal and Peterson, 2015). Tapentadol is a weak opioid and the conducted trials were often methodologically poor. There is no sufficient data to support the use of newer drug tapentadol over other opioids. Other opioids have been longer on the market and are less expensive.

Dextropropoxyphene has a long half-life in older people and has been associated with a significant risk of side effects (Becquemont et al., 2014). It was observed that dextropropoxyphene market withdrawal had no consequences on the intensity or impact of chronic pain in older patients.

Pethidine has an active metabolite that accumulates in older people with kidney failure (Wilder-Smith, 2005).

There is insufficient data on the use of methadone as an analgesic in the older population with cancer. Because of its pharmacological characteristics it must be used by trained personnel. Several recommendations are proposed for its use as an analgesic in the treatment of cancer pain in the older age (Taberna et al., 2014).

Morphine, fentanyl, oxycodone, or buprenorphine are still the preferred evidence-based choices for add-on opioid therapy for older people or frail patients (Veal and Peterson, 2015).

## Pharmacokinetics of opioids

Aging can bring some changes in the pharmacokinetics of drugs. Prescribing pain medications within the older population requires the skill of a very knowledgeable physician to navigate through the numerous variables (e.g., physiologic changes, comorbid conditions, multiple medications) that make the older patients a heterogeneous and complex population to treat (Pergolizzi et al., 2008; Vezmar Kovačević et al., 2014). The pathophysiologic profile of older adults significantly changes with age. Decline in organ function, particularly the renal and hepatic functions, which are critical in the clearance of ingested substances, dictates the pharmacokinetic properties of many drugs (Tumer et al., 1992).

Absorption can be altered because of decreased gastrointestinal blood flow and increase of gastric pH. With aging, there are changes in body composition: increase in adipose tissue, decrease in body mass, and decrease in body water, what can have influence on the distribution of drug (Chau et al., 2008). Also, decreasing hepatic blood flow can decrease metabolism of drugs. The first pass of metabolism opiates can be reduced in older age (Tegeder et al., 1999). Also, decreased glomerular filtration rate can lead to accumulation of metabolites of opioids and to more adverse effects.

Protein binding could represent an important site of interaction (Table 1). Most of the drugs bind to albumin, but buprenorphine binds to alpha and beta globulins. As a result, drug–drug interactions related to protein binding for this drug is small (Pergolizzi et al., 2008).

**Table 1:** **Pharmacokinetics of selected opioids**.

**Opioid**	**Plasma protein binding (%)**	**Volume of distribution (l/kg)**	**Half-life (*t*_1∕2_)**	**Phase I metabolism**	**Phase II metabolism**	**Major metabolites**
Morphine	30	245	3	CYP3A	UGT2B7	Morphine-3-glucuronide (M3G)—neuroexcitatory effects
						Morphine-6-glucuronide (M6G)—analgesic effect
Hydromorphone	20	91.5	2.6		UGT2B7	Hydromorphone-3-glucuronide
					UGT1A3	
Fentanyl	84	4	3.5	CYP3A		Norfentanyl-inactive
Tramadol	20	2.6	6.7	CYP3A		Nortramadole-inactive
				CYP2D6		*O*-desmethyltramadol-active
Oxycodone	45	2.6	3.7	CYP3A	UGT2B7	Oxymorphone
				CYP2D6		Noroxycodone
Methadone	85–90	1–8	8–59	CYP3A		No active metabolites
Codeine	7–25	3–6	3–4	CYP3A	UGT2B7	Codeine-6-glucuronide (C6G)
				CYP2D6		Morphine
						Norcodeine
Tapentadol	20	540	4	CYP2D6	UGT1A9	No active metabolites
				CYP2C9	UGT2B7	
				CYP2C19		
Pethidine	40	3–5	4	CYP2D6		Norpethidine (neurotoxic, analgesic effect)
				CYP3A		
				CYP2C19		
Dextropropoxyphene	70–80	16	15	CYP3A		Nordextropropoxyphene
Buprenorphine	96	188–335	3–4	CYP3A4 CYP2C8	UGT1A1 UGT1A3 UGT2B7	Norbuprenorphine Buprenorphine-3-glucuronide Norbuprenorphine-3-glucuronide

Most of opioids are metabolized by CYP P450 isoenzymes with a variability that is largely determined by genetic polymorphisms (Gianni et al., 2009; Table 1). CYP polymorphisms may account for high rates of side-effects or diminished efficacy in affected patients. Oxycodone and tramadol, are metabolized mostly by CYP 2D6, and buprenorphine is metabolized by CYP 3A4. CYP is among the principal pathways of drug metabolism for several drugs, and this could represent a relevant problem in older patients treated with complex polypharmacological regimens (Pergolizzi et al., 2008). A reduction of ~20% in the metabolism of CYP 2D6 substrates was observed in older population (Dorne et al., 2002; O'Connell et al., 2006), whereas such a finding has not been confirmed for the CYP 3A subfamily. Certain pharmacokinetics parameters of some opioids are shown in Table 1.

## Adverse effects of opioids

Adverse effects of opioids are well-known: nausea, constipation, urinary retention, pruritus, respiratory depression, cardiovascular, and central nervous system side effects (Vučković et al., 2004). The analysis of the impact of opioid treatment on terminal cancer patients on consciousness, appetite and thirst was performed in one randomized controlled trial. Morphine, fentanyl, oxycodone or codeine were used for palliative treatment in these patients. Adverse event incidence rates were 25% for constipation, 23% for somnolence, 21% for nausea, 17% for dry mouth, and 13% for vomiting, anorexia, and dizziness. Adverse effects as asthenia, diarrhea, insomnia, mood change, hallucinations, and dehydration occurred at incidence rates of 5% and below (Wiffen et al., 2014).

The principle “start low, go slow” is highly recommended when treatment with opioids is initiated. Profile of the adverse effect of opiates is similar for all age groups, although older patients are at greater risk due to comorbidities and interactions with other medications (Chau et al., 2008). German guideline for long-term use of opioids recommends a critical check after 3 months in non-cancer pain and in case of dosing over 120 mg morphine equivalents is advisable, especially for older persons. Cognitive effects and sedation occur frequently in chronic non-malignant pain populations receiving long-term opioid therapy and are among the most common reasons patients discontinue opioid use (Dhingra et al., 2015). Suggested recommendation from The European Association of Palliative Care (EAPC) Research Network for management of opioid side effects include the following: dose reduction, symptomatic management of the adverse effects using drugs targeting the symptoms, opioid rotation, and switching the route of administration (Chau et al., 2008). The risk of falls and fractures, which is a special concern in older patients may be reduced by using longer acting opioids instead of short acting opioids (Schuler and Grießinger, 2015).

The difference between younger and older population who used fentanyl-based intravenous patient-controlled analgesia (IV-PCA) after surgery was evaluated in one retrospective analysis of 10,575 patients. It was concluded that a larger proportion of older patients required rescue antiemetics despite lower incidence of postoperative nausea and vomiting compared to the younger (Koh et al., 2015). Also, there was a single report of opioid (fentanyl)-induced muscle rigidity causing life-threatening respiratory compromise in a 79 old male patient (Dimitriou et al., 2014).

A further study focused on various opioids and their therapeutic benefits and risks in older persons, particularly levorphanol, tapentadol, and transdermal buprenorphine. Levorphanol was found to be similar to methadone regarding efficacy, but without pharmacokinetic and other interactions of methadone. Tapentadol was associated with significantly less gastrointestinal distress and constipation. Transdermal buprenorphine had less risk for the toxicities associated with conventional opioids and with compliance benefits (Atkinson et al., 2013).

## Interactions of opioids

Drug interactions are common with opioids and represent an important safety issue. Clinical studies indicate that 80% of patients over the age of 65 years have at least one chronic condition while 50% have more than one. The same age group uses between 2 to 6 prescribed medications and 1 to 3.4 non-prescription medications daily (Stewart and Cooper, 1994; Larsen and Martin, 1999). The risk of adverse drug reactions increases with the number of medications used (Larsen and Martin, 1999). Older people may be particularly susceptible to unintentional misuse of prescription opioids because high rates of polypharmacy and a high prevalence of chronic pain. Results of one study showed that population rates of misuse of prescription opioids were higher in older than in younger adults and those rates among the older ages increased each year. Also, it was reported that serious medical outcomes among the older population followed an increasing linear trend (West and Dart, 2016; some serious opioid outcomes are shown in Table 2).

**Table 2:** **Serious opioid interaction outcomes**.

Opioids				
+				
Alcohol	Sedatives	Antihistamines	Antipsychotics	Muscle relaxants
		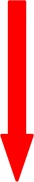		
Additive/synergistic interaction				
		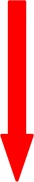		
Respiratory depression				
Hypotension				
Sedation				
Coma				
Death				


A general list of medications that many older patients are commonly prescribed include cardiovascular drugs (thiazide diuretics, beta-blockers, angiotensin-converting enzyme inhibitors); antidiabetic drugs (metformin, sulfonylureas, thiazolidinediones); bronchodilators, and steroid inhalers such as fluticasone/salmeterol, tiotropium, albuterol; non-sedating antihistamines, and steroid, nasal sprays (Maggiore et al., 2010). The most common interactions of opioids with other medications are shown in Table 3.

**Table 3:** **Interactions of opioids with other drugs**.

Stimulants CNS	Increase of ventricular arrhythmias (*methadone+atomoxetine*)
Antiepileptic drugs	Dextropropoxyphene increases the effect of carbamazepine
Tricyclic antidepressants	Increase of sedative effects of opioids
	Increase of toxic effects (convulsions, e.g., *tramadol*)
	Inhibition of morphine glucuronidation
Monoamine oxidase inhibitors (MAOIs)	Excitation and depression of CNS (*pethidine, tramadol*)
Selective serotonin reuptake inhibitors (SSRIs)	Toxic CNS effects (convulsions, *tramadol*)
Anticoagulants	Increase of anticoagulant effects (*dextropropoxyphene, tramadol*)
Antiarrhythmics	Merphine can increase the concentration of esmolol in plasma
Drugs affecting gastrointestinal tract	Metoclopramide and domperidone antagonize the gastrointestinal effects of opioid analgesics
	Cimetidine inhibits the metabolism of opioids
	Ranitidine decreases the concentration of morphine and conversion to active metabolites
Antibacterial drugs	Opioids decrease the concentration of ciprofloxacin
	Erythromycin increases opioid concentration
	Rifampicin decreases opioid concentration
Antiviral drugs	Metadone can increase the concentration of zidovudine
	Ritonavir can increase the concentration of opioid analgesics (*fentanyl, petidine, dextropropoxyphene*)
Antifungal drugs	Vorikonazol increases concentration of methadone and alfentanyl
	Ketoconazole increases the concentration of opioid analgesics

## Conclusion

The pharmacologically-guided approach to the treatment of moderate to severe pain in older persons requires a systematic understanding of the pharmacokinetics and pharmacodynamics of analgesics. An understanding of the changes associated with aging is very important for clinical use of opioids. The absence of randomized controlled trials in the older population emphasizes the importance of relying on clinical judgment. While a number of opioids may be commonly used in older people, some representatives of this class have an unacceptable risk to frail older patients. It is important to follow guidelines for use of opioids in older age.

## Author contributions

MP, KSV, SV, BM, DS, ND, RS, AV, LP, AJ, NC—design of the work; or the acquisition, analysis, or interpretation of data for the work.

### Conflict of interest statement

The authors declare that the research was conducted in the absence of any commercial or financial relationships that could be construed as a potential conflict of interest.
